# Using “Enzan No Metsuke” (Gazing at the Far Mountain) as a Visual Search Strategy in Kendo

**DOI:** 10.3389/fspor.2020.00040

**Published:** 2020-04-29

**Authors:** Takaaki Kato

**Affiliations:** Faculty of Environment and Information Studies, Keio University, Fujisawa, Japan

**Keywords:** eye movements, Kendo, peripheral vision, visual pivot, expertise, Enzan no Metsuke

## Abstract

In Kendo (Japanese fencing), “Enzan no Metsuke” is an important *Waza* (technique) that is applied by expert Kendo fighters. It involves looking at the opponent's eyes with “a gaze toward the far mountain,” taking in not only the opponent's face but also his or her whole body. Over the last few decades, a considerable number of studies on visual search behaviors in sport have been conducted. Yet, there are few articles that examine visual search behaviors in combat sports, such as martial arts. This study aimed to analyze the visual search strategies used by expert Kendo fighters through sparring practices to discuss what “Enzan no Metsuke” is under experimental, but natural (*in situ*), conditions. Ten experts, 10 novices, and one *Shihan* (a master of Kendo) participated in this study. The fighters wore a mobile eye tracker and faced a real opponent. They were instructed to do the following in five different sessions: prepare themselves, practice their offense and defense techniques, and fight in a real *Shiai* (match). The results indicated differences in the visual search strategies between the *Shihan*, experts, and novices. The *Shihan* and experts fixated on their opponent's eyes or head region most of the time and adopted a visual search strategy involving fewer fixations of longer duration. Conversely, novices set their eyes mainly on the opponent's *Shinai* (sword). Only the *Shihan* always looked at the opponent's eyes, even during the preparation, offense, and defense sessions. *Shihan* and experts set their “visual pivot” on the opponent's eyes quietly, even when the opponent tried to attack with the *Shinai*. Novices, however, moved their eyes up and down based on the influence of their opponent's movements. As these results indicate, novices tried to search for detailed information about their opponent and processed visual information depending on their focal vision, whereas *Shihan* and experts absorbed information not from their opponent's eyes but from their entire body by utilizing their peripheral vision; this means that *Shihan* and experts could see an opening or opportunity and react instantaneously by using “Enzan no Metsuke.”

## Introduction

In most sporting situations, players have to make quick, accurate decisions under severe spatial and temporal constraints. Successful sporting performance requires efficient and accurate execution of movement patterns and the ability to perceive important information from a complex and constantly changing environment. For instance, players of a ball game, like soccer, or basketball, must act on the visual information presented by the ball, their opponents, and their teammates (Williams et al., 1999). The process of obtaining information accurately and rapidly from selected areas of the visual display is known as a “visual search.” Reports reveal that expert players do not move their eyes randomly; rather, they adopt visual search patterns based on deliberate perceptual strategies (e.g., Bard and Fleury, 1976; Williams et al., 1999; Vickers, 2007). Over the last few decades, a considerable number of studies examining visual search behaviors in sport have been conducted. Mann et al. (2007) conducted a meta-analysis on experts' perceptual-cognitive skills in their chosen sport. Results revealed that they tended to rely on fewer fixations of longer duration compared with less-skilled players or novices.

Conversely, Mann et al. (2009) showed that expert football (soccer) players had superior decision-making skills with more search fixations of shorter duration when viewing aerial video footage as opposed to a player's perspective. In this situation, it appears that skilled athletes optimize their performance through searching more locations (Williams and Jackson, 2019). Recently, Kredel et al. (2017) reported that eye-tracker studies have increased and that field studies conducted under *in situ* conditions, or those with larger degrees of external validity, contributed to developing an understanding of players' visual information input in sport-related tasks.

Currently, few studies have examined visual search behaviors in combat sports such as boxing, kung fu, karate, and judo (Ripoll et al., 1995; Williams and Elliott, 1999; Piras et al., 2014; Milazzo et al., 2016b; Hausegger et al., 2019). Ripoll et al. (1995) showed that expert boxers adopted a more efficient search pattern compared to non-experts and tended to maintain foveal fixation as a “visual pivot” on central regions of the opponent's body while using their peripheral vision to acquire information from the hands and feet regarding the initiation of an attack. Williams and Elliott (1999) also reported that expert karate fighters exhibited superior anticipation compared to non-experts when experiencing varying levels of anxiety and that they “anchored” their fovea on the central regions of their visual display while using their peripheral vision to monitor their opponent's limb movements. Thus, these articles suggest a correlation between the level of expertise and the fighter's visual search strategy. Milazzo et al. (2016b) reported that expert karate fighters spent more time fixating on their opponent's head and the torso with a low search rate, as opposed to novices, who spent more time fixating on the pelvis and the front hand of their opponent with a high search rate. Similarly, expert judo fighters used a search strategy involving fewer fixations of longer duration and spent more time fixating on the lapel and face compared to their novice counterparts (Piras et al., 2014). Expert kung fu fighters (like Tae Kwon Do fighters) attack mostly with their legs anchored and their gaze focused at the lower region of their opponent to monitor the relevant cues for kicking attacks (Hausegger et al., 2019).

Considering these studies, fixating on the opponent's head and trunk region and anchoring the gaze on a specific location to use their peripheral vision for picking up relevant cues, like a suspected punch or a kick, is a functional visual strategy in contact sports.

**Figure 1:**
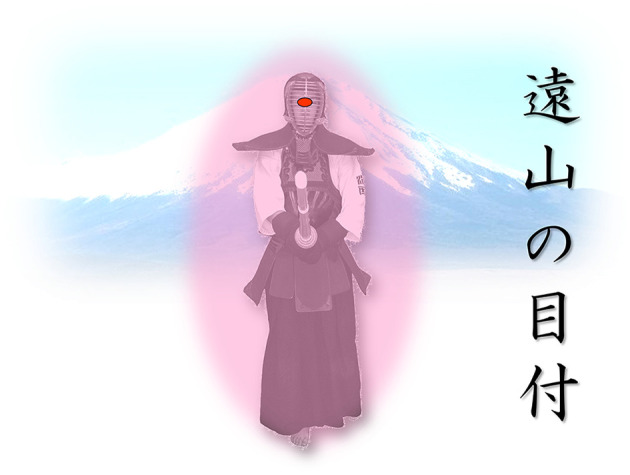
The concept of “Enzan no Metsuke.” It is said that “Enzan no Metsuke” is one of the most important *Waza* whereby fighters look at their opponent with a gaze toward the mountains in the distance, taking in not only their opponent's face but their whole body as well.

The *Waza* technique is used in Kendo (also known as the Way of the Sword and the art of Japanese Samurai Swordsmanship) and is especially applied by expert Kendo fighters. In Kendo, the sequence of perception to *Waza* is described as *Ichi gan* (first for eye), *Ni soku* (second for feet), *San tan* (third for abdomen or center), and *Shi riki* (fourth for power). Sight is the first element of any technique, and the way Kendo fighters watch their opponents is crucial to the success of their attack. “Metsuke” means the point of observation or the way of seeing. It is said that “Enzan no Metsuke” (gazing at the far mountain) is one of the most important *Waza* whereby fighters look at their opponent with a gaze toward the mountains in the distance, taking in not only their opponent's face but their whole body as well (Figure 1). Salmon (2013), a Kendo fighter, explains this concept in the following way “If we stare at the target we are going to strike, we give our opponent obvious notices of our intention. If we look just at his or her face to try to understand their next action, we may miss the signals he gives when he starts to move hands or feet. If we look just at feet or hands, we can be easily tricked by movement designed to get our attention. If we look at the point of the *Shinai*, there is even more chance that we may be fooled by a feint” (p. 46). Therefore, the gaze should see everything without focusing on any one point. Fighters are instructed in “Enzan no Metsuke” to fix their gaze on their opponent's eyes and utilize their peripheral vision in order to pick up information from the whole-body movements of their opponents frequently in practice, and they try to practice while thinking about it. However, studies have yet to analyze the visual search behavior of “Enzan no Metsuke” from the viewpoint of empirical scientific research.

In this paper, I explain the visual search behavior of “Enzan no Metsuke” by investigating the differences in the visual search strategies between expert Kendo fighters and novices, especially including *Shihan*, who is a Kendo master under the live Kendo practice and competitive *in situ* conditions. Analysis of how they use their eyes and the visual pivot technique to gain informative and efficient visual information will also be included. Our main hypothesis was that expert Kendo fighters adopt a visual search strategy that involves fewer fixations of longer duration and the use of peripheral vision based on the findings of previous studies regarding visual search behaviors in combat sports (Ripoll et al., 1995; Williams and Elliott, 1999; Piras et al., 2014; Milazzo et al., 2016b; Hausegger et al., 2019).

## Materials and Methods

### Participants

Twenty Kendo fighters in university and one Shihan (a master of Kendo) were recruited as participants. The expert group contained 10 fighters from the university team (mean age = 20.4 years; SD = 1.4) with an average of 13.7 years (SD = 3.2) of prior Kendo experience. They had been awarded the fourth Dan, which is a relatively high rank in a grading system that consists of six basic grades called Kyu (sixth to first) and eight advanced grades called Dan (first to eighth). The novice group contained 10 fighters (mean age = 20.8 years; SD = 1.3) with an average of 3.9 years (SD = 1.4) of prior Kendo experience only in physical education class but who had no prior competitive level experience. Only one Shihan, aged 65 years, who was awarded the eighth Dan and Hanshi title, which is the highest attainable rank, was included. He had mastered and completed the principle of the Sword and had outstanding knowledge of Kendo. Therefore, he participated in this study as a special reference and was not examined using statistical analysis. The experimental protocol was approved by the institutional ethics committee of Keio University SFC, and the tenets of the Declaration of Helsinki were observed. Written informed consent was obtained from each participant, and all fighters reported normal vision or corrected-to-normal vision.

### Apparatus

Visual search behaviors were recorded using a lightweight eye movement registration system EMR-9 (NAC Image Technology Inc., Tokyo, Japan). The system utilized the pupil and corneal reflex method at 60 Hz. Its precision was <0.1 degrees in both the horizontal and vertical directions. Data were stored on the SD card in the recording unit and then transferred onto a computer. For the analysis of performance during each session, all behaviors of participants were recorded using an external video camera situated at a distance of 5 m away from the center of the court.

### Procedure

**Figure 2:**
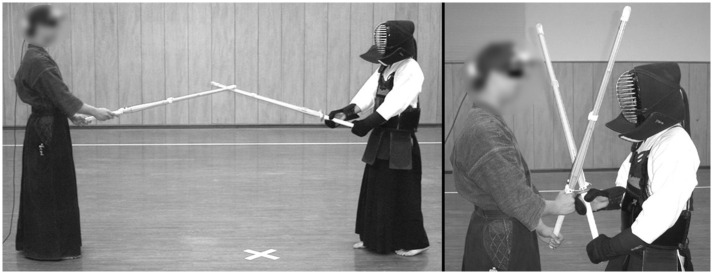
Experiment scenes (**Left**, common distance ~2.5 m between fighters in S1, S3, S4, and S5; **Right**, *Tsubazeriai* distance, which is extremely close, the swords were tangled, and the average interpersonal distance was ~1 m in S2).

The task and constraints of the experimental conditions were explained to the participants before each trial. It was confirmed before the experiments that all participants had knowledge of “Enzan no Metsuke” regardless of whether they had mastered it in the right manner or not, and they were not instructed about “Enzan no Metsuke” during the experiments. They performed an individual, generalized warm-up, and the EMR-9 system was fitted. Initially, the system was calibrated and validated with a nine-point reference grid presented ~2.5 m in front of the fighters, which is the average interpersonal distance between two fighters (Yamamoto et al., 2016) (Figure 2). All participants competed against the same one opponent from the expert group who behaved in a manner similar to a real match situation. At the beginning of each trial, participants and the opponent were precisely positioned face-to-face in an upright posture. They then competed in a real match situation for a few minutes. Gaze accuracy was maintained by having participants fixate on constant reference points before and after every trial, so that a recalibration could be performed, if necessary.

Five different sessions (S) which were similar tasks to those in usual sparring practices for Kendo fighters, were conducted, and all of the data that were analyzed in each session were the same length of time for all participants: Preparation (S1; 18 s) was performed after the fighters took a standing bow and *sonkyo* (crouching down with the sword) at a distance. They then stood up and came closer to take a posture with the cutting edge of the sword facing toward their opponent with the sword tips crossing slightly. It was not required for them to do any additional attacks or defenses. S1 lasted about 20 s, and the data of last 18 s were analyzed because the fighters tended to move their eyes outside of the opponent's body areas, which included useless data such as no fixation during the time they stood up, walked to the opponent, and took a posture in the initial part of the session. Close-contact (S2; 18 s) was performed with the fighters in the *Tsubazeriai* situation (close together with swords tangled, and the average interpersonal distance was ~1 m at this time) (Figure 2) and trying to push their opponent back to create another chance for them to attack. Fighters were required to strike their opponent's *Men* (Kendo head armor) once while quickly moving backward to make a proper space. Each attack was repeated until success, and the number of attempts was counted. S2 lasted from about 20–30 s, and the data of the last 18 s were analyzed. Offense practice (S3; 12 s) was performed when fighters were required to strike their opponent's *Men, Do* (side trunk covered by a stomach and chest protector), and *Kote* (lower forearm covered by a gauntlet), once for each. Each attack was repeated until success, and the number of attempts was counted. It took about 5 s for each attack, and the data of the last 4 s were analyzed. Defense practice (S4; 12 s) was performed three times when the fighters started to defend against their opponent's *Kote* attack. The number of blocked attacks was counted. It took about 5 s for each attack, and the data of the last 4 s were analyzed. *Shiai* (S5; 60 s) was performed when the fighters competed in almost the same manner as a real match (barring attacks to the *Men*). The session actually lasted from 1.5 to 4 min from the time the referee called *Hajime* (begin) until the referee called *Yame* (stop) when one of the fighters was awarded two points. The data of the last 60 s were analyzed because some of the experts already got two points after <2 min from *Hajime*. By reference to previous research (Nakamura et al., 2014), the number of offensive techniques (striking an opponent before the opponent initiates an attack), counter techniques (striking an opponent after rendering the opponent's attacks ineffective), defensive techniques (defending against the opponent's attack), and awarded points (scored if the participant struck an opponent accurately) were counted.

### Data Analysis

The number of attempts in S2 and S3, the number of blocked attacks in S4, and the number of offensive, counter, and defensive techniques and awarded points in S5 were evaluated as the performance during each session. The between-group differences across each of these measures were analyzed separately using Welch's *t*-test and Cohen's *d* effect size measures for each task. Also, a success rate, which was the number of successes divided by number of attempts, was calculated for each session.

**Figure 3:**
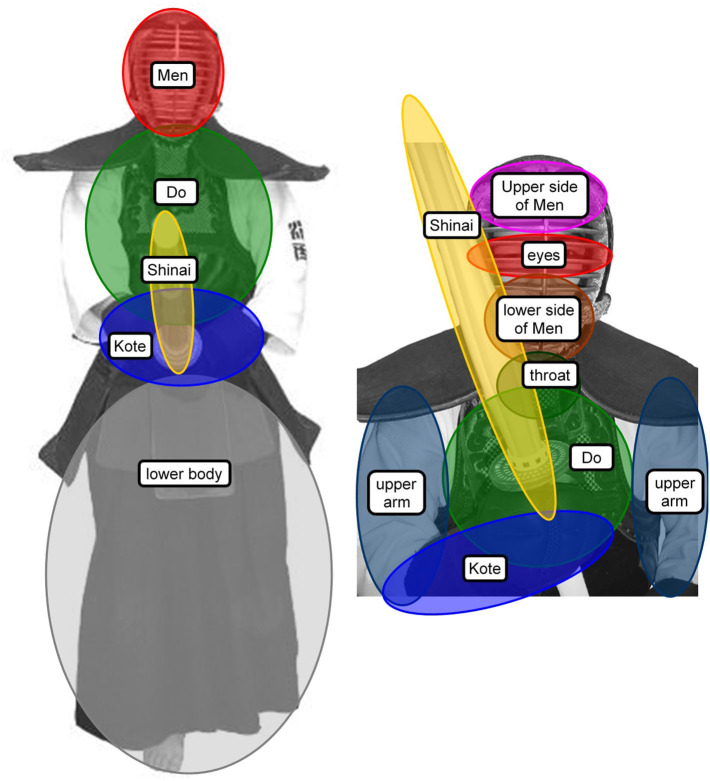
The area of fixation location chosen for the study (**Left**, the five areas considered the whole body in S1, S3, S4, and S5; **Right**, a more specific eight areas in the *Tsubazeriai* distance in S2).

Eye movement data encoded by the EMR-9 system were analyzed frame-by-frame to obtain the visual search rate and percentage viewing time values. The fighters' visual fields were divided into specific areas to derive the fixation location: *Men, Do, Shinai* (a sword), *Kote*, lower body, or other, which was included to account for those fixations that did not fall within any of the above areas. The visual angle of the height of the opponent (181 cm) held at normal interpersonal distance is ~39.8 degrees. The area of *Kote*, which is the smallest area of interest, subtends ~3.4 degrees. It was only during S2, when the fighters were in the *Tsubazeriai* situation, that location was more specifically categorized to the upper or lower side of the *Men*, middle of the *Men* (eyes), throat, *Do*, upper arm, *Kote, Shinai*, or other, because the fighters, particularly the novices, tried to move their eyes over more specific locations at close-up interpersonal distance in S2 (Figure 3). The visual angle of the height of the opponent at this distance is ~84.3 degrees. The area of the middle of the *Men* (eyes), which is the smallest area of interest, subtends ~3.2 degrees. Fixation was defined as the period when the eye remained stationary within 1 degree of movement tolerance for a period >99 MS (three video frames) (Vickers, 2007). All trials were taken into consideration for the mean number of fixations, mean fixation duration, and mean number of fixation locations per trial as search rate. The mean number of fixation locations is the average number of locations fixated on according to the categorized areas of the display.

For further analysis, the percentage of viewing time, which is the total amount of time fighters spent viewing each area of the visual display, included the above mentioned fixation locations during all trials (Ward et al., 2002; Roca et al., 2011). The between-group differences across each of these search rate measures (number of fixations, fixation duration, and number of fixation locations) were analyzed separately using Welch's *t*-test and Cohen's *d* effect size measures for each task. The percentage viewing time was analyzed using a mixed two-way ANOVA for each task in which the fixation locations were the within-subject factors and groups were the between-subject factors. Mauchly's sphericity test was used to validate the ANOVA, and partial eta-squared values ($\eta_{\text{p}}^{2}$) were used to reflect the strength/magnitude related to the effect of these factors. The sources of any interactions were examined using *post-hoc* comparisons with Shaffer's modified sequentially rejective Bonferroni procedure. The significance level was set at α = 0.05.

## Results

### Performance

The number of attempts, the number of blocked attacks, and the number of offensive, counter, and defensive techniques and awarded points between expert and novice groups are shown in Table 1. There were significant group differences for the number of attempts in S2, *t*_(12.13)_ = 2.63, *p* = 0.022, *d* = 1.18, and S3, *t*_(13.17)_ = 4.84, *p* < 0.002, *d* = 2.16. Similarly, there were significant group differences for the number of blocked attacks in S4, *t*_(17.86)_ = 7.96, *p* < 0.001, *d* = 3.56. Additionally, there were significant group differences in S5 for the number of counters; *t*_(16.82)_ = 4.19, *p* < 0.001, *d* = 1.87, the number of defensive techniques, *t*_(18)_ = 2.42, *p* = 0.025, *d* = 1.08; and the number of awarded points, *t*_(13.75)_ = 3.54, *p* < 0.003, *d* = 1.58. Experts and *Shihan* performed better than novices in all sessions, with a high success rate.

**Table 1:** Mean (SD) number of attempts in S2 and S3, number of blocked attacks in S4, and number of offensive, counter, and defensive techniques and awarded points in S5 between expert and novice groups.

	***Shihan***	**Expert**	**Novice**
**S2: close-contact**			
Number of attempts	1	1.2 (0.42)	2.1 (0.99)
Success rate	100.0%	83.3%	47.6%
**S3: offense task**			
Number of attempts	3	3.3 (0.67)	5.5 (1.27)
Success rate	100.0%	90.9%	54.5%
**S4: defense task**			
Number of blocked attacks	3	2.5 (0.53)	0.7 (0.48)
Success rate	100.0%	83.3%	23.3%
**S5:** ***Shiai*** **(match)**			
Number of offensive techniques	7	7.1 (1.20)	6.3 (1.42)
Number of counter techniques	3	2.5 (1.08)	0.7 (0.82)
Number of defensive techniques	3	3.1 (0.74)	3.9 (0.74)
Number of awarded points	2	1.2 (0.79)	0.2 (0.42)

*The success rate is the percentage of required success divided by the number of attempts*.

### Search Rate

The mean number of fixations, mean fixation duration, and mean number of fixation locations between expert and novice groups on each task are shown in Table 2. There were significant group differences for the mean number of fixations per trial in all sessions: S1, *t*_(17.16)_ = 15.77, *p* < 0.001, *d* = 7.06; S2, *t*_(10.6)_ = 6.85, *p* < 0.001, *d* = 3.06; S3, *t*_(13.74)_ = 3.81, *p* < 0.01, *d* = 1.70; S4, *t*_(13.22)_ = 7.72, *p* < 0.001, *d* = 3.45; and S5, *t*_(9.72)_ = 18.61, *p* < 0.001, *d* = 8.32. Similarly, there were significant group differences for the mean fixation duration per trial in all sessions: S1, *t*_(9.03)_ = 4.03, *p* < 0.01, *d* = 2.07; S2, *t*_(9.08)_ = 3.89, *p* < 0.01, *d* = 1.74; S3, *t*_(12.37)_ = 2.81, *p* = 0.02, *d* = 1.26; S4, *t*_(9.40)_ = 4.68, *p* < 0.01, *d* = 2.09; and S5, *t*_(9.03)_ = 4.53, *p* < 0.01, *d* = 2.03. Additionally, there were significant group differences for the mean number of fixation locations per trial in all sessions: S1, *t*_(9)_ = 5.71, *p* < 0.001, *d* = 2.56; S2, *t*_(17)_ = 7.80, *p* < 0.001, *d* = 3.49; S3, *t*_(14.31)_ = 2.60, *p* = 0.021, *d* = 1.16; S4, *t*_(16.29)_ = 5.58, *p* < 0.001, *d* = 2.50; and S5, *t*_(12.91)_ = 5.02, *p* < 0.001, *d* = 2.24. In most cases, experts employed a less exhaustive visual search strategy involving fewer fixations of longer duration and a smaller number of fixation locations than the novice group. Additionally, the results of the *Shihan* data showed very few numbers of fixation and longer fixation durations in all sessions, even though the data were not analyzed statistically.

**Table 2:** Mean (SD) number of fixations, mean fixation duration, and mean number of fixation locations per trial across groups in all sessions (trial duration).

	**Number of fixations**	**Fixation duration (ms)**	**Number of fixation locations**
**S1: preparation (18 s)**			
*Shihan*	2.00	8,999	1.00
Expert	5.60 (2.76)	3,199 (1,691.94)	2.30 (1.49)
Novice	23.20 (2.20)	717 (72.79)	5.00 (0.00)
**S2: close-contact (18 s)**			
*Shihan*	2.00	8,999	1.00
Expert	5.60 (2.76)	3,112 (1,995.63)	2.70 (0.82)
Novice	26.40 (9.20)	649 (134.77)	6.00 (1.05)
**S3: offense task (12 s)**			
*Shihan*	4.00	2,633	1.00
Expert	6.40 (1.84)	1,678 (740.80)	3.10 (0.74)
Novice	11.10 (3.45)	958 (326.57)	3.80 (0.42)
**S4: defense task (12 s)**			
*Shihan*	2.00	6,049	1.00
Expert	4.30 (2.87)	2,808 (1,498.79)	1.50 (0.53)
Novice	20.00 (5.75)	566 (222.18)	3.10 (0.74)
**S5:** ***Shiai*** **(match, 60 s)**			
*Shihan*	11.00	5,166	3.00
Expert	12.70 (3.30)	4,459 (2,759.19)	2.50 (1.08)
Novice	112.00 (16.55)	501 (105.33)	4.40 (0.52)

### Percentage Viewing Time

**Figure 4:**
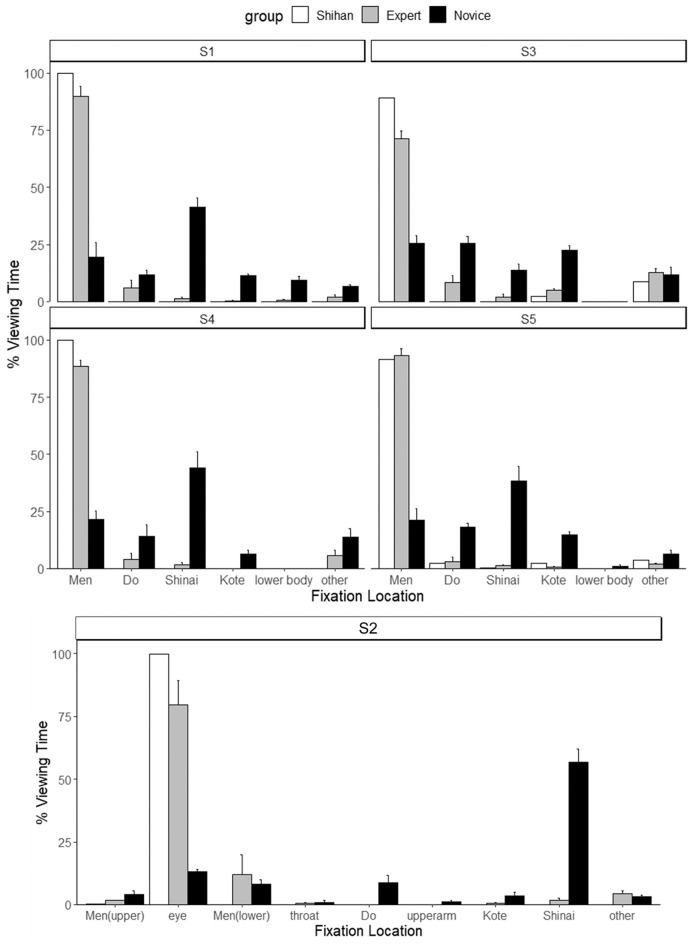
Mean (SD) percentage viewing time per fixation location which is categorized in Figure 3.

Figure 4 illustrates the differences in the percentage of viewing time for both groups in all sessions. A violation of the sphericity assumption for mixed measures ANOVA was found in S1, S2, S3, and S5 using Mauchly's sphericity test, S1, *W* = 182.79, *p* < 0.001; S2, *W* = 1,362.99, *p* < 0.001; S3, *W* = 166.06, *p* < 0.001; and S5, *W* = 189.79, *p* < 0.001. Therefore, degrees of freedom were corrected using the Greenhouse–Geisser epsilon for suggested violation (S1, ε = 0.28; S2, ε = 0.19; S3, ε = 0.50; S5, ε = 0.34). Significant differences were observed for the Group × Fixation location interaction in S1, *F*_(1.41, 25.3)_ = 70.98, *p* < 0.001, $\eta_{\text{p}}^{2}$ = 0.80; S2, *F*_(1.53, 27.55)_ = 36.47, *p* < 0.001, $\eta_{\text{p}}^{2}$ = 0.67; S3, *F*_(2.49, 44.9)_ = 43.61, *p* < 0.001, $\eta_{\text{p}}^{2}$ = 0.71; S4, *F*_(5, 90)_ = 50.25, *p* < 0.001, $\eta_{\text{p}}^{2}$ = 0.74; and S5, *F*_(1.68, 30.3)_ = 81.40, *p* < 0.001, $\eta_{\text{p}}^{2}$ = 0.82. *Post-hoc* tests revealed that experts spent a greater percentage of the time fixating on *Men, F*_(1, 18)_ = 81.69, *p* < 0.001, $\eta_{\text{p}}^{2}$ = 0.82, whereas novices spent more time watching the *Shinai, F*_(1, 18)_ = 96.29, *p* < 0.001, $\eta_{\text{p}}^{2}$ = 0.84; *Kote, F*_(1, 18)_ = 530.57, *p* < 0.001, $\eta_{\text{p}}^{2}$ = 0.97; and lower body, *F*_(1, 18)_ = 25.56, *p* < 0.001, $\eta_{\text{p}}^{2}$ = 0.59 in S1. Similarly, in S2, experts spent significantly more time fixating on the eye, *F*_(1, 18)_ = 45.59, *p* < 0.001, $\eta_{\text{p}}^{2}$ = 0.72, while novices spent more time on *Do, F*_(1, 18)_ = 8.60, *p* = 0.009, $\eta_{\text{p}}^{2}$ = 0.32; upper arm, *F*_(1, 18)_ = 8.31, *p* = 0.001, $\eta_{\text{p}}^{2}$ = 0.32; *Kote, F*_(1, 18)_ = 5.67, *p* = 0.03, $\eta_{\text{p}}^{2}$ = 0.24; and *Shinai, F*_(1, 18)_ = 105.64, *p* < 0.001, $\eta_{\text{p}}^{2}$ = 0.85. In S3, experts focused more on *Men, F*_(1, 18)_ = 100.38, *p* < 0.001, $\eta_{\text{p}}^{2}$ = 0.85, and novices focused more on *Do, F*_(1, 18)_ = 15.33, *p* < 0.01, $\eta_{\text{p}}^{2}$ = 0.46; *Shinai, F*_(1, 18)_ = 18.17, *p* < 0.001, $\eta_{\text{p}}^{2}$ = 0.50; and *Kote, F*_(1, 18)_ = 54.16, *p* < 0.001, $\eta_{\text{p}}^{2}$ = 0.75. In S4, experts fixated more on *Men, F*_(1, 18)_ = 201.72, *p* < 0.001, $\eta_{\text{p}}^{2}$ = 0.92, whereas novices fixated more on *Shinai, F*_(1, 18)_ = 33.05, *p* < 0.001, $\eta_{\text{p}}^{2}$ = 0.65, and *Kote, F*_(1, 18)_ = 16.58, *p* < 0.001, $\eta_{\text{p}}^{2}$ = 0.48. Furthermore, experts spent significantly more time viewing *Men, F*_(1, 18)_ = 153.16, *p* < 0.001, $\eta_{\text{p}}^{2}$ = 0.89 in S5, while novices spent more time viewing *Do, F*_(1, 18)_ = 32.56, *p* < 0.001, $\eta_{\text{p}}^{2}$ = 0.64; *Shinai, F*_(1, 18)_ = 34.95, *p* < 0.001, $\eta_{\text{p}}^{2}$ = 0.66; and *Kote, F*_(1, 18)_ = 80.84, *p* < 0.001, $\eta_{\text{p}}^{2}$ = 0.82. Irrespective of the session, the expert group spent significantly more time fixating on the *Men* or eye in S2. In contrast, the novice group spent more time fixating on the *Shinai* in most cases. Additionally, the *Shihan* set his line of sight on his opponent's *Men* or eye in all sessions.

## Discussion

The aim of this study was to elucidate the visual search behavior of expert and novice Kendo fighters under *in situ* conditions, to scientifically demonstrate “Enzan no Metsuke” during a real match of Kendo. We hypothesized that experts demonstrate superior performance with a more efficient visual search strategy involving fewer fixations of longer duration and the use of peripheral vision compared with their novice counterparts. The results of this study reveal that experts set their eyes on the opponent's “eye,” which is inside the *Men* most of the time during Kendo practice, and they employed a less exhaustive visual search strategy involving fewer fixations of longer duration and a smaller number of fixation locations. In contrast, the novices tended to search for detailed information on the opponent under the influence of their opponent's sword and body movements, and the results of the search rate showed that they made a great number of short fixations and used a large number of fixation locations.

As mentioned previously, looking into the eyes of the opponent has been emphasized as one of the most important *Waza*, not only in Kendo but in almost all of *Budo* (martial arts in Japan) as well. Ozawa (1997) stated that “you should not focus your eyes on one point; rather, you should focus on the body as a whole, taking your partner's eyes as a central point” (p. 41). It is considered that “Enzan no Metsuke” allows Kendo fighters to use peripheral vision and pick up advanced cues from the whole-body movements of their opponents (Figure 1). In this study, detailed peripheral visual processing of fighters from eye movement data could not be examined, because of the limitation of *in situ* experimental situations. Still, the results of performance analyses revealed that experts could act or react quickly and accurately with a high success rate (Table 1). It is assumed that experts could utilize peripheral vision properties “focusing” on the body as a whole while setting their line of sight on the opponent's eye; therefore, they made a better prediction by picking up relevant cues from distal body areas such as an opponent's sword, arm, and even foot.

The present findings agree with those reported by Piras et al. (2014), who revealed that expert judo fighters spent a great percentage of their time fixating their gaze on the central regions of their opponent's upper body, primarily the lapel and the face. Williams and Elliott (1999) and Milazzo et al. (2016b) indicated that expert karate fighters “visually anchored” their attention on the head and torso, while Ripoll et al. (1995) showed that expert French boxers maintained foveal fixation mainly on the central regions of their opponent's body as a “visual pivot,” while using their peripheral vision to monitor their opponent's limb movements. Such “visual pivot” or “visual anchor” strategies have been reported in other sports like baseball batting (Kato and Fukuda, 2002), soccer penalty kicking (Savelsbergh et al., 2002; Piras and Vickers, 2011), one-on-one defense in soccer (Nagano et al., 2004), golf putting (Naito et al., 2004), and volleyball reception (Vansteenkiste et al., 2014), which indicates that these strategies are not only confined to combat sports. More recently, Vater et al. (2019) proposed the definition and operationalization possibilities of three different gaze strategies: the “foveal spot,” the “gaze anchor,” and the “visual pivot.” While the concepts of pivots or anchors have been newly defined, it is beneficial to discuss these suggestions, and further evidence to support these concepts should be collected.

When comparing the results of this study to previous studies, it must be noted that the visual search strategies of experts may depend on the subjective task demand required of each player relative to his or her particular skills. Hagemann et al. (2010) examined expert épée fencers' eye movements under laboratory settings with filmed video stimuli incorporating temporal and spatial occlusion. The study revealed that experts fixated particularly on the upper trunk but shifted their eye movements to neighboring body regions when the upper trunk was occluded. Furthermore, the viewing percentage distribution results showed that experts fixated more on their opponent's weapon (26%) in the lower trunk region (30%) than the upper trunk region (about 15%) and head (1%). It is significant to note that the entire body is a valid target in épée fencing, and it is therefore inferred that experts needed to attend not only to the proximal region of their opponents' bodies but also to the distal region as well. Similarly to that study, Hausegger et al. (2019) reported that expert kung fu (Qwan Ki Do) athletes focused more on their opponent's head, whereas Tae Kwon Do athletes mainly attacked their legs and anchored their gaze on their opponent's lower region to monitor the relevant cues if kicking attacks were solely expected. In contrast, Kendo fighters are only allowed to attack their opponent's *Men, Do*, and *Kote*, which are all above the hip; hence, it is assumed that they focus their gaze on the eyes of their opponent. Again, it is said that “Enzan no Metsuke” is the *Waza* whereby fighters look at their opponent's eyes with a gaze toward the mountains in the distance, taking in not only their opponent's face but their whole body as well. A recent study has demonstrated that fixating at a far target would contribute to faster reaction and that the effect is specific to the focus location in the peripheral visual field (Kokubu et al., 2018). Although the distance of expert Kendo fighters' focus location was not measured in this study, it is considered that they tried to focus on a distant place through the opponent's eye and utilize peripheral vision with “Enzan no Metsuke,” so that they showed better performance with a high success rate during all sessions.

Notable findings indicate that expert Kendo fighters adopt a visual search strategy involving fewer fixations of longer duration than novices who make a greater number of short fixations. These results are consistent with what has been found in previous research in judo (Piras et al., 2014) and karate (Williams and Elliott, 1999; Milazzo et al., 2016b). Furthermore, Milazzo et al. (2016a) showed that karate fighters changed their visual search behavior by focusing on fewer locations for a longer duration, and experienced fighters improved decision-making accuracy if they received video-based implicit perceptual-motor training. When analyzing mean fixation duration (Table 2), this study revealed that Kendo experts fixated for an average of 1,678–4,459 ms on each location in all the sessions, which is much longer than the 328 ms recorded for karate (Williams and Elliott, 1999), 760 ms for judo (Piras et al., 2014), 1,026 ms in karate (Milazzo et al., 2016b), and 2,423 ms in French boxing (Ripoll et al., 1995). Ultimately, the *Shihan* showed a much longer fixation duration than the experts in most of the sessions. A possible explanation for this might be that Kendo fighters tend to keep their fighting distance while waiting for the right opportunity to attack without any dynamic movements, which is referred to as *Maai*. The state of that behavior sometimes looks like freezing; therefore, their eyes also seemed to move quietly. Conversely, during open ball-play sports like soccer, experts typically employed a visual search strategy involving more fixations of shorter duration in the display (e.g., not only fixating on the ball but the positions and movements of the other players as well) because their environment changes dynamically (e.g., Ward et al., 2002; Roca et al., 2011). These exhaustive and dynamic visual search behaviors are thought to underpin the superior anticipation and decision-making skills of experts (Williams et al., 1999).

In target tasks such as a basketball free throw or darts, the “quiet eye” (QE) is utilized. The QE is defined as the final fixation, or tracking gaze, on a specific object or location in the period before the unfolding of the final movement that is critical for a successful performance. It has been argued that an earlier and longer QE duration is a characteristic of elite performers (Vickers, 2007, 2016). In this study, sessions were not terminated by each attack; rather, they lasted till a specific ending time (see *Procedure* section). Therefore, expert Kendo fighters spontaneously kept their eyes on the eyes of their opponent to utilize their peripheral vision to pick up relevant cues from the distal body regions, even after their own attack movement had unfolded. Their eyes moved “quietly” but fixated on a specific target only before the unfolding of the final movement.

One limitation of this study is the lack of records of any additional responses, such as pushing keys or verbal reports. For example, it is not clear how widely they could pick up information from peripheral visual areas, how far they focused, or what they thought during tasks. The ultimate goal of the study was considered to be the examination of the natural, visual behaviors of Kendo fighters during *in situ* conditions from an ecological validity viewpoint. Thus, we decided not to include any additional responses during the sessions, because gaze and movement behaviors function differently depending on the experimental task constraints selected for empirical investigations (Dicks et al., 2010). Moreover, the natural motor response of the participants should be favored over verbal or button-press responses (Kredel et al., 2017).

The *Shihan*, aged 65 years, displayed ultimate visual behaviors by employing effective and stable visual search activities, and it is assumed that he utilized his peripheral vision to pick up relevant cues from the opponent's distal body areas Vater et al. (2019). Any additional tests for functional abilities were not conducted at this time, so it is unclear what specific abilities the *Shihan* possesses. Nevertheless, he performed very well, with a high success rate (Table 1) during all sessions. In this study, he participated as a special reference and was not examined using statistical analysis. Recent research has demonstrated that older martial arts athletes (judo and karate) perform better than non-athletes of the same age in the investigation of peripheral vision and perceptual asymmetry tasks (Muiños and Ballesteros, 2014), and physical activities including martial arts can improve peripheral vision properties in older individuals [for a review, see (Muiños and Ballesteros, 2018)]. It was invaluable that *Shihan* who are awarded the eighth *Dan* and *Hanshi* title, which is the highest attainable rank in Japan (with over 40 years prior kendo experience, but acceptant rate is below 1%), participated in this study, and the data regarding his visual behavior is extremely valuable, not only for science but also in the practical domain.

## Conclusion

The purpose of the present study was to clarify the visual search strategies of expert Kendo fighters through sparring practices to analyze the application of the concept of “Enzan no Metsuke” under *in situ* experimental conditions. Results revealed that experts, especially *Shihan*, set their eyes on their opponent's eyes with a gaze toward the mountains in the distance to utilize their peripheral vision. Additionally, they adopted a visual search strategy that involves fewer fixations of longer duration. These results reveal that the “visual pivot” strategy can be regarded as a behavior of “Enzan no Metsuke”; however, further research should be conducted to investigate the relationships between peripheral vision and motor control in more detail.

## Data Availability Statement

The datasets generated for this study are available on request to the corresponding author.

## Ethics Statement

The studies involving human participants were reviewed and approved by Institutional Ethics Committee of Keio University SFC. The patients/participants provided their written informed consent to participate in this study.

## Author Contributions

TK contributed the conception, design, and conduct of the study, and also wrote the whole manuscript.

## Conflict of Interest

The author declares that the research was conducted in the absence of any commercial or financial relationships that could be construed as a potential conflict of interest.
